# Influence of wear parameters on friction performance of A356 aluminum – graphite/ granite particles reinforced metal matrix hybrid composites

**DOI:** 10.1016/j.heliyon.2019.e01770

**Published:** 2019-06-10

**Authors:** Tirlangi Satyanarayana, Putti Srinivasa Rao, M.Gopi Krishna

**Affiliations:** a Dept. of Mechanical Engg, Andhra University, Visakhapatnam, India; b Dept. of Mechanical Engg, Acharya Nagarjuna University College of Engineering, Guntur, 522510, India

**Keywords:** Materials science

## Abstract

In this work aluminium-silicon alloy (A356) is strengthened with blends of granite and graphite particles using stir casting technique. Granite particulates, an industrial waste generated while cutting granite stones is used along with graphite particulates which has excellent lubricant properties. The reinforcements are mixed in different weight proportions, to base alloy at 2 and 4 weight percentages. Wear rate and friction for the fabricated composites and alloy is recorded during each sliding test The dry sliding wear of the over different loads with a track diameter of 60 mm and sliding times of 15, 30 and 45 minutes respectively. The techniques of ANOVA and Taguchi are used to study the influence of wear parameters on the friction coefficient of composites by using “MINITAB 18” software with quality characteristics chosen as smaller is better.

## 1 Introduction

Due to the improved mechanical and tribological properties, aluminium metal matrix composites (AMMC's) got great importance and became a part of aviation and automobile/vehicle industries. The main fundamental demand from the automobile industry is to find advanced materials, which are capable of reducing fuel utilization and vehicle discharges [1]. The aluminium metal matrix composites are advanced materials which consists of two phases i.e. matrix and the reinforcement for example, SiC, B_4_C, TiC, Al_2_O_3_ are hard reinforcements to fabricate composite materials [2]. Most of the ceramic reinforcements are chemically in tune with the aluminium metal and forms perfect bonding so that the properties like thermal conductivity, workability specific strength, stiffness, wear resistance, fatigue and corrosion will be improved. The enhanced properties lead to aluminium metal matrix composites to play a vital role in various applications like cylinder liners, barrels automobile components like piston, connecting rods, brakes and other power transmission elements. The friction and wear performance of various hybrid metal matrix composites and the important parameters which influence the wear and friction like applied load, sliding distance and reinforcement percentage were reported by many authors [3, 4, 5, 6, 7]. Taguchi approach and analysis of variance techniques were used by many authors to find the most affecting parameters on wear and friction. Baradeswaran *et al.* studied the wear behavior of A7075/graphite/Al_2_O_3_ hybrid composites and reported that the friction coefficient of 7075 alloy decreases with 5 percent addition of graphite and up to 8 percent addition of Al_2_O_3_ [8]. The friction coefficient of graphite particulates reinforced in pure aluminium up to 5 wt.% decreases with increase in sliding velocity and sliding distances is reported by Rajesh *et al* [9]. From different studies it is revealed that various field/process/wear parameters influencing wear, coefficient of friction of AMC's. The wear, frictional performance is not in homogeneous pattern for all composites with reference to change in wear parameters. In the present study the friction and wear behaviors of 356 aluminium alloy reinforced with graphite and granite dust particles were analyzed by taguchi and ANOVA techniques at different field conditions.

## 2 Materials & methods

### 2.1 Material fabrication

In the present investigation, aluminium based hybrid metal matrix composites containing various combinations and weight percentages of granite and graphite particulate reinforcements of 53μm were successfully synthesized by eddy method. The matrix materials used in this study was Al–Si alloy (A356) whose chemical composition was shown in Table 1.

**Table 1:** Chemical composition of Al–Si alloy, wt. %.

Si	Mg	Cu	Fe	Ti	Al
6.5	0.4	0.05	0.09	0.06	Balance

**Fig. 1:**
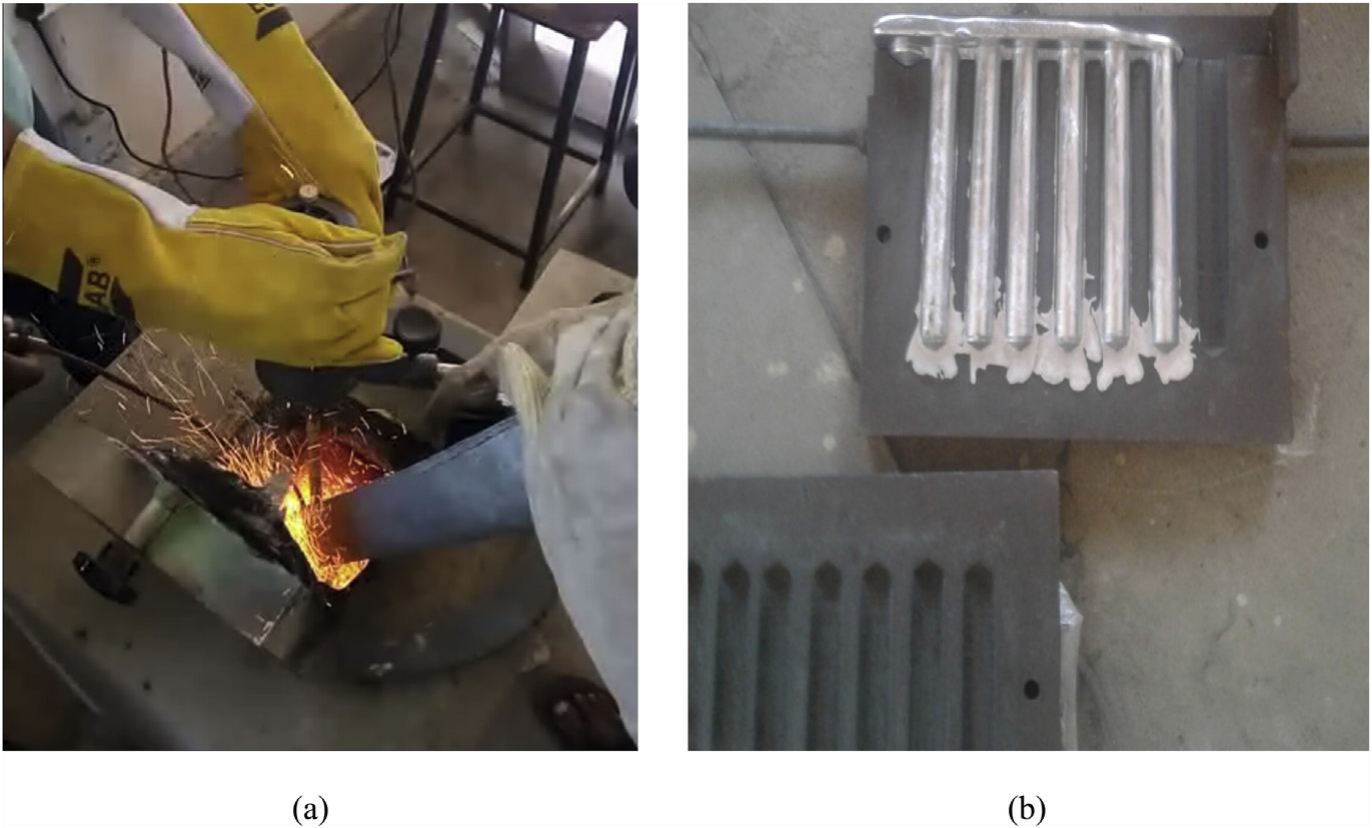
(a) Experimental set up of stir casting (b) Alloy and composite fingers in mould.

Al–Si alloy is melted in a graphite crucible which is placed inside a muffle furnace. The stir casting setup is shown in Fig. 1(a) Once the required temperature is attained i.e. (770 °C) a pool was created. The preheated particulates of granite and graphite at were poured into the melt in different weight proportions of 2% graphite and 2% granite in first case and by keeping the graphite reinforcement as constant the granite weight percentage is increased to 4%. A conical shaped object made of tin is used to ensure the continuous and smooth flow of the particles which will be added exactly in the vortex. For comparison purpose pure graphite powder is reinforced without adding granite. Argon gas is to be shielded around the melt to prevent oxidation. The fingers casted in Grey cast iron mould is depicted in Fig. 1 (b)

### 2.2 Wear tests

**Fig. 2:**
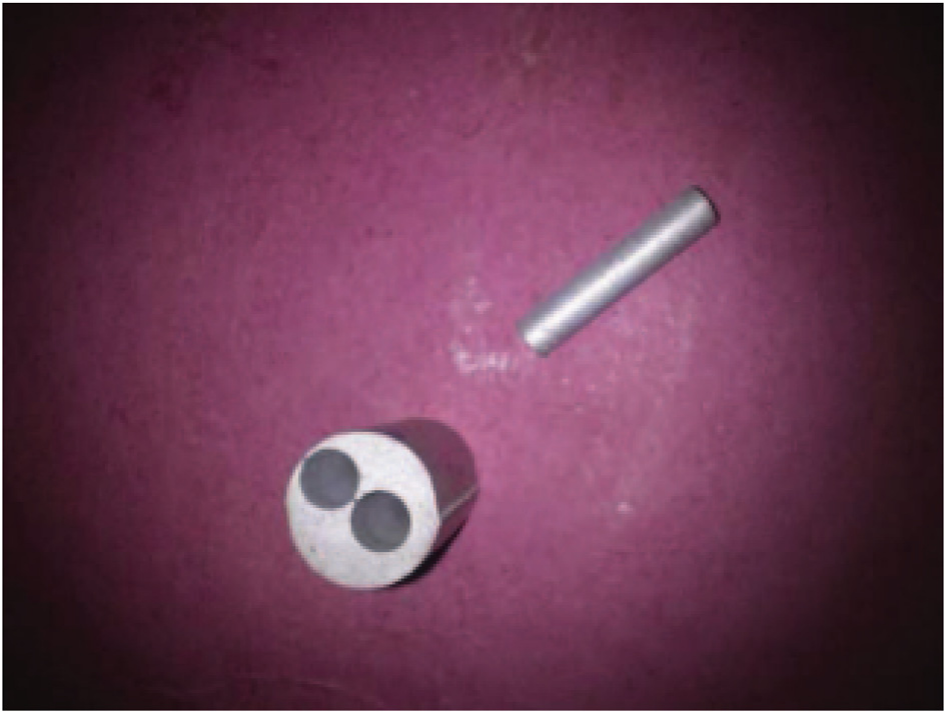
Wear samples machined by wire cut EDM.

The dry sliding wear behavior of A356/Graphite/Granite dust particulates reinforced composite specimens were investigated using a pin-on- disc wear testing equipment (Model: Ducom TR- 20 LE) with data acquisition system. The required specimens were machined to 3 mm diameter and 30 mm length and metallographically polished and cleaned with acetone as shown in Fig. 2. The specimens were pressurized against a rotating EN31 steel disc (hardness 65 HRC) at room temperature 25 °C and 65% relative humidity. The friction coefficient of A356 hybrid composite continuously monitored and recorded separately during each sliding test.

For conducting wear tests three parameters of load, reinforcement percentage and sliding time and three levels are planned for the work. The first two are process parameters and third one is material dependent parameter shown in Table 2.

**Table 2:** Parameters and levels.

Level	Load (Kgf) (L)	Reinforcement (Wt %) (R)	Sliding Time (Min) (T)
1	0.5	Base alloy 356	15
2	1	(2%Granite)	30
3	1.5	(2%Graphite+4% Granite)	45

### 2.3 Experimental values of coefficient of friction

The coefficients of friction for various combinations of parameters such as load percentage of reinforcement and sliding time are obtained as per L_27_ orthogonal array. An average value of coefficient of friction is considered from three trails of each set of experimental parameters is given in Table 3. A validation test is conducted to check the accuracy of the experiments done with taguchi approach and found that the experimental value is very nearer to the predicted value which validates the results shown in Eq. (1).

**Table 3:** Values of Coefficient of friction.

Load/Reinforcement	Base alloy 356			2% Granite Reinforcement			2% Graphite+4% Granite		
	*15 Min*	*30 Min*	*45 Min*	*15 Min*	*30 Min*	*45 Min*	*15 Min*	*30 Min*	*45 Min*
0.5 kgf	0.289	0.185	0.163	0.265	0.213	0.204	0.263	0.255	0.128
1.0 kgf	0.322	0.275	0.239	0.282	0.271	0.245	0.283	0.261	0.131
1.5 kgf	0.344	0.301	0.264	0.284	0.283	0.255	0.291	0.276	0.199

Optimum conditions for coefficient of friction (COF) = L1R3T3.

The experimental values at L1R3T3 is 0.128.

Predicted value at optimal condition

(1) $$\begin{array}{l} {\text{COF}}_{\text{Predicted}}\;=\;\text{μ}+\text{(L}1-\;\text{μ)}+\text{(R}3-\;\text{μ)}+\text{(T}3-\;\text{μ)}\\ \;\;\;\;\;=\text{L}1+\text{R}3+\text{T}3-2\;\text{μ}\\ \;\;\;\;\;=0.2183+0.2319+0.2031-2\times 0.2508\\ \;\;\;\;\;=0.1517 \end{array}$$

### 2.4 Results and discussion on microstructures of composites

**Fig. 3:**
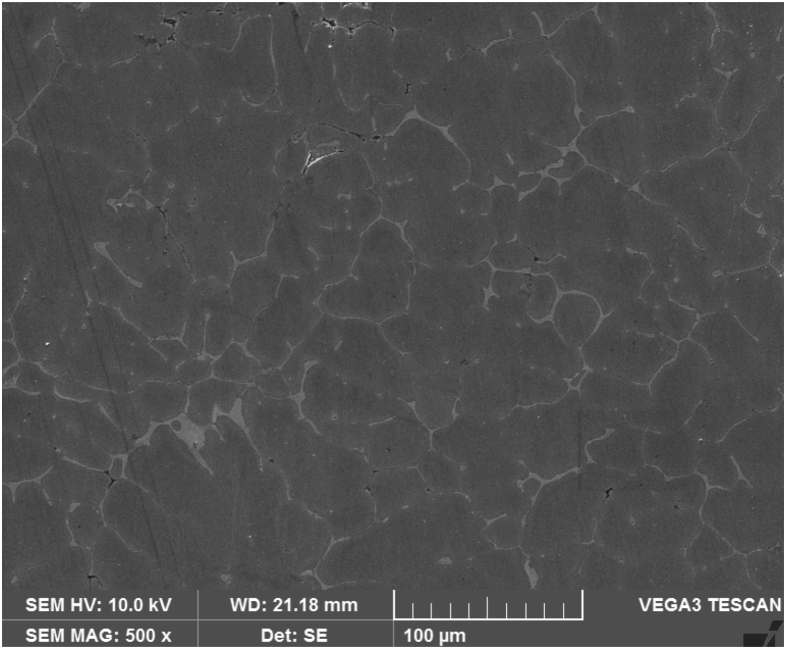
Al–Si base alloy.

**Fig. 4:**
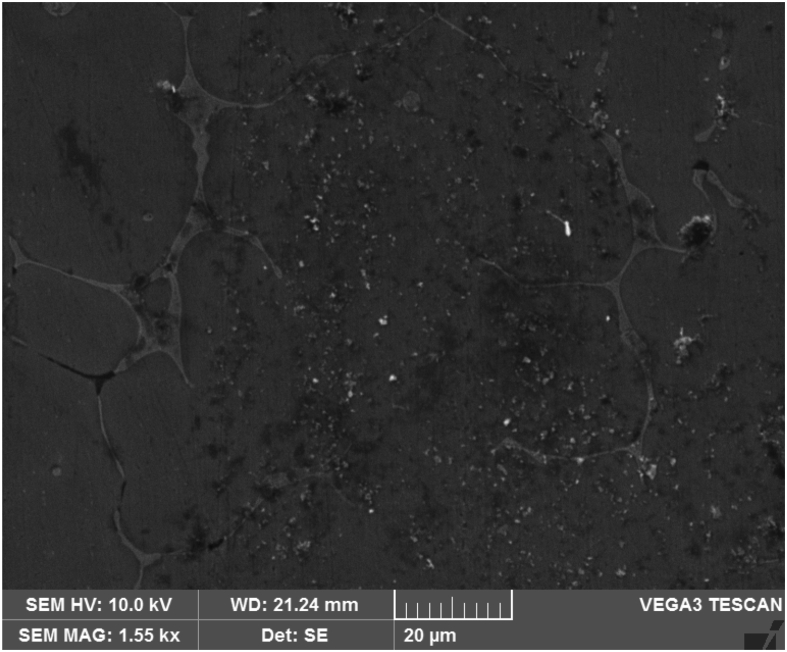
Al–Si base alloy with 2 % granite reinforcement.

**Fig. 5:**
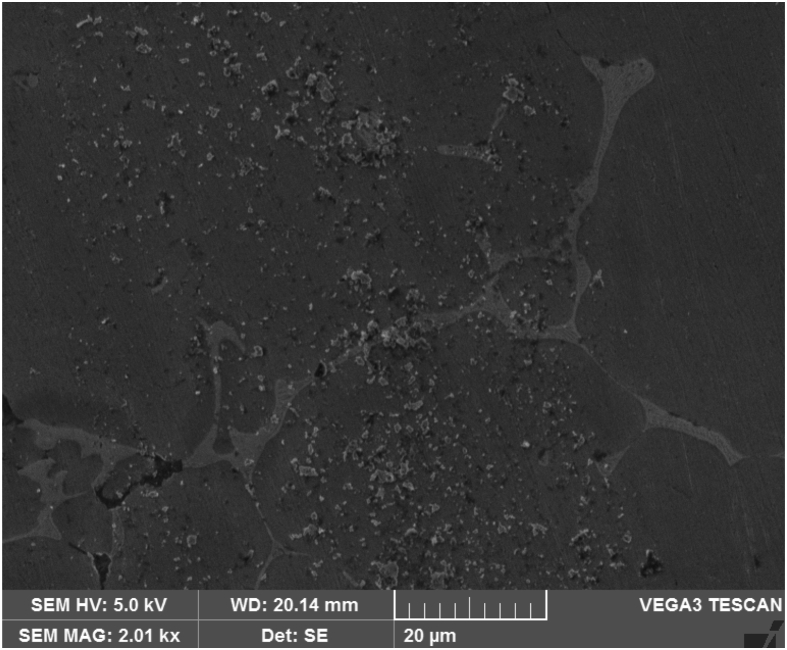
Al–Si reinforced with graphite and granite dust showing uniform distribution.

Fig. 3 shows the SEM micrograph of Al–Si base alloy a primary Silicon like needle structures are identified with inter dendritic regions and the granite particles are well distributed in the matrix as shown in Fig. 4 and it is evident that the uniform distribution of both granite and graphite particles depicted in Fig. 5.

## 3 Results & discussion

Statistical software MINITAB 18 is used to measure the frictional coefficient results in three phases.

1 Signal to noise ratio calculations for parameters which influence the coefficient of friction.
2 ANOVA analyses are used to know the percentage of contribution for each parameter on coefficient of friction.
3 Normal plot study is done to find the standardized effects of parameters and its level. 3D surface plots are drawn to estimate coefficient of friction with reference e to any two process parameters.

### 3.1 Results for signal to noise ratio

The results obtained from the experiments are converted into signal-to- noise ratio to estimate the quality characteristics. Coefficient of friction is the response with the objective function that smaller one is better. The influencing parameters such as percentage of reinforcement, applied load, sliding time on coefficient of friction are analyzed. The measured values and S/N ratiaos for coefficient of friction of composites are given in Table 4.

**Table 4:** Factor's S/N ratiao table.

S.No	Load (Kgf) (L)	Reinforcement (Wt%) (R)	Sliding Time (min) (T)	Coefficient of friction	S/N Ratio
1	0.5	0	15	0.289	10.78204
2	0.5	0	30	0.185	14.65657
3	0.5	0	45	0.163	15.75625
4	0.5	2	15	0.265	11.53508
5	0.5	2	30	0.213	13.43241
6	0.5	2	45	0.204	13.8074
7	0.5	2 + 4	15	0.263	11.60089
8	0.5	2 + 4	30	0.255	11.8692
9	0.5	2 + 4	45	0.128	17.8558
10	1	0	15	0.322	9.842883
11	1	0	30	0.275	11.21335
12	1	0	45	0.239	12.43204
13	1	2	15	0.282	10.99502
14	1	2	30	0.271	11.34061
15	1	2	45	0.245	12.21668
16	1	2 + 4	15	0.283	10.96427
17	1	2 + 4	30	0.261	11.66719
18	1	2 + 4	45	0.131	17.65457
19	1.5	0	15	0.344	9.268831
20	1.5	0	30	0.301	10.42867
21	1.5	0	45	0.264	11.56792
22	1.5	2	15	0.284	10.93363
23	1.5	2	30	0.283	10.96427
24	1.5	2	45	0.255	11.8692
25	1.5	2 + 4	15	0.291	10.72214
26	1.5	2 + 4	30	0.276	11.18182
27	1.5	2 + 4	45	0.199	14.02294

### 3.2 Analysis of results for signal to noise ratio

The ranking of process parameters and its influence based on mean of coefficient of friction obtained for its level is given in Table 5. The ranking of process parameters and its influence based on S/N ratio for the coefficient of friction are obtained for its level is given in Table 6.

**Table 5:** Response table for frictional coefficient.

Level	Load (L) Kgf	Reinforcement (R) Wt.%	Sliding time (T) Min
1	0.2183	0.2647	0.2914
2	0.2566	0.2558	0.2578
3	0.2774	0.2319	0.2031
Delta	0.0591	0.0328	0.0883
Rank.	2	3	1

**Table 6:** Response table for coefficient of friction S/N ratios (smaller the better).

Level	Load (L) Kgf	Reinforcement (R) Wt.%	Sliding time (T) Min
1	13.48	11.77	10.74
2	12.04	11.90	11.86
3	11.22	13.06	14.13
Delta	2.26	1.29	3.39
Rank	2	3	1

### 3.3 Effects of parameters on coefficient of friction

**Fig. 6:**
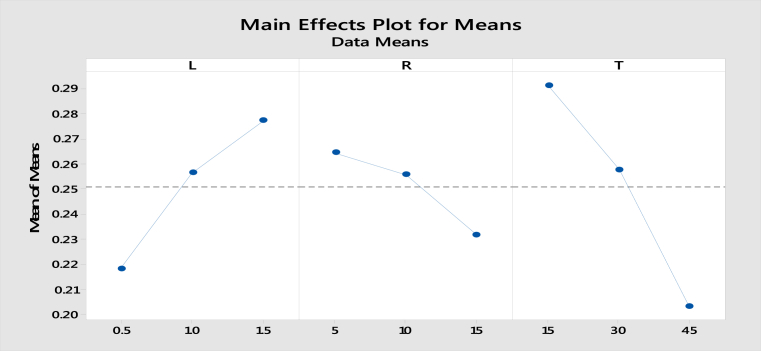
Main effects plot for means –COF.

**Fig. 7:**
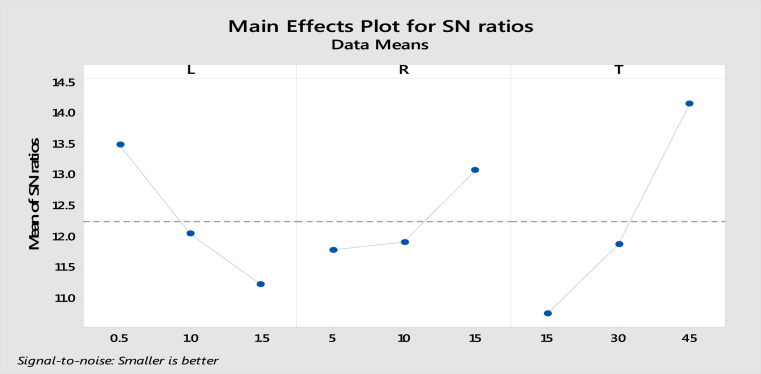
Main effects plot for S/N ratio –COF.

The influence of each parameter independently on the outcome of frictional coefficient is mentioned in main effects plot as shown in Fig. 6. The main effects plot for data means for all parameters by considering them independently on the outcome of S/N ratio is shown in Fig. 7.

How much each parameter can influence the outcome of coefficient of friction is interpreted using main effects plot. The optimum or best values can be easily read from those plots. The influence of each parameter can be seen in the form of slope of main effects plot. More tendency means higher is the influence. The sliding time is the highest influential factor follows load and percentage reinforcement.

**Fig. 8:**
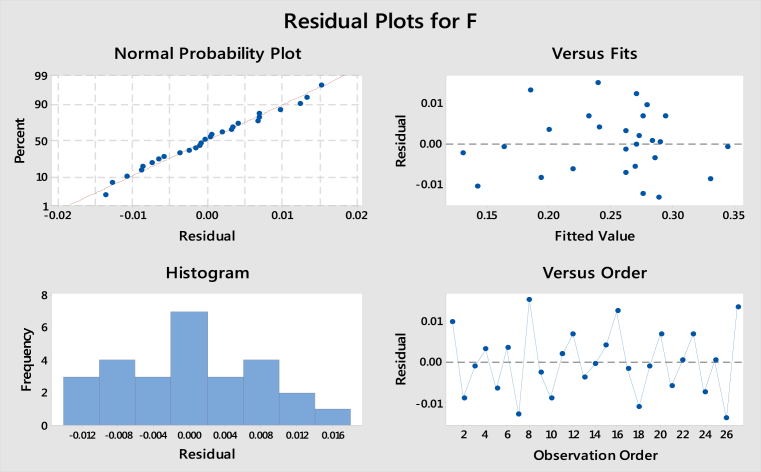
Residual plots for coefficient of friction.

The residual plots for coefficient of friction are inferred from taguchi analysis of MINITAB 18 Software is shown in Fig. 8. The normal probability plot shows the experimental values are very close to fitted line, hence the trueness of ranks of influential parameters holds good. Residual verses fits are scattered in a range of ±0.01, lager experimental values are close to negligible deviation. The level of a factor with highest signal to noise ratio is the optimal level. Apart from the category of the quality characteristic a greater S/N ratio corresponds to a better performance.

#### 3.3.1 Effect of load on coefficient of friction

The coefficient of friction increases with increasing load. The graphite and granite particles are pulled during dry sliding and the silicon dioxide (SiO_2_) layers present in granite particles which are influenced by the temperature over the surface of the contact, as the load increase from 0.5 Kgf to 1.5 Kgf the delamination of partially oxidized hard silicon layer occurs, forming scratches and groves on the pin surface. Umanath *et al* [10] reported a similar relation, where the coefficient of friction is decreased with increased load. It can also be incidental that the coefficient of friction is higher in composites with coarse particulates. At the outline, the coefficient of friction is decreased with an increase in the percentage of granite dust, load, and speed and increased with an increase in particle size.

#### 3.3.2 Effect of sliding time on coefficient of friction

The coefficient of friction decreases with increasing sliding time, as the sliding time increases with plastic deformation of the surface occurred leading to adhesion of the pin surface on to the disc and it leads to more material removal and this material forms a mechanically mixed layer called tribo layer which acts as a barrier or lubricant between two surfaces decreasing the coefficient of friction as the sliding time increases.

#### 3.3.3 Effect of reinforcement on coefficient of friction

M.K.Surappa et al [11] observed that by increasing finer flyash reinforcement content in 2024 alloy exhibited superior properties in composites. The coefficient of friction is decreased with increased reinforcement due to the formation of solid lubricant film as tribo surface. The possibility for small granite particles to be pulled out from the matrix was greater, hence resulting in an increased wear volume.

### 3.4 ANOVA analysis

The analysis of Variance (ANOVA) analysis is used to analyze the experimental results influenced by the parameters load, sliding time, and weight percentage of reinforcement on the coefficient of friction. For a level of significance at 5%, i.e., the level of confidence 95%, to study the percentage contribution of each factor on coefficient of friction. ANOVA analysis results are given in Table 7. The sixth Column in Table 7 shows the percentage contribution (P) of each factor on total variation indicating their degree of influence on the coefficient of friction of the composite.

**Table 7:** Analysis of Variance for coefficient of friction.

Source	DF	SS	MS	F	P	% of Contribution
L	2	0.016174	0.008087	39.99	0.000	24.87
R	2	0.005172	0.002586	12.79	0.003	7.95
T	2	0.035774	0.017887	88.45	0.000	55.01
L*R	4	0.003389	0.000847	4.19	0.040	2.60
L*T	4	0.001652	0.000413	2.04	0.181	1.27
R*T	4	0.009959	0.002490	12.31	0.002	7.65
Error	8	0.001618	0.000202	–	–	0.62
Total	26	0.073739	–	–	–	100

It is observed from the results obtained in Table 7, that the sliding distance is the most considerable parameter having the highest percentage contribution (55.01%) on the coefficient of friction of composites followed by load (24.87%) and finally Reinforcement (7.95%). Hence sliding time is an important control factor to be taken into consideration of coefficient of friction during wear process followed by load and percentage of reinforcement (weight percentage of graphite and granite Particles). From Table 8, as R square value is about 95% the results from the ANOVA model analysis are holds good.

**Table 8:** Model summary.

S	R-sq	R-sq (adj)
0.0142205	97.81%	92.87%

### 3.5 Normal plot analysis of the parameters on coefficient of friction

**Fig. 9:**
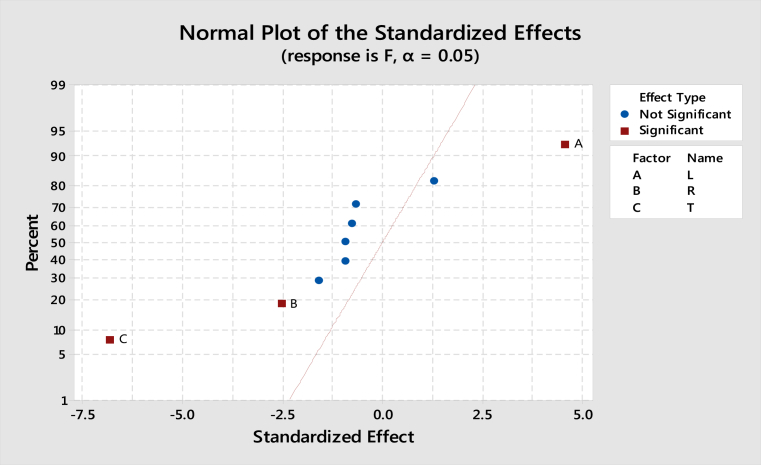
Normal plot of the standard effects.

Fig. 9 shows the normal plot of the various parameters. Significant terms are indicated with brown colored rectangle, where as non significant terms indicated with blue colored circle. Both terms T,R are left side to the normal plot indicates, that those contribute in the decreasing of coefficient of friction upon its increase. The term L is on the right side of normal plot, hence it indicates coefficient of friction increases upon its increase.

### 3.6 Three dimentional surface plots

**Fig. 10:**
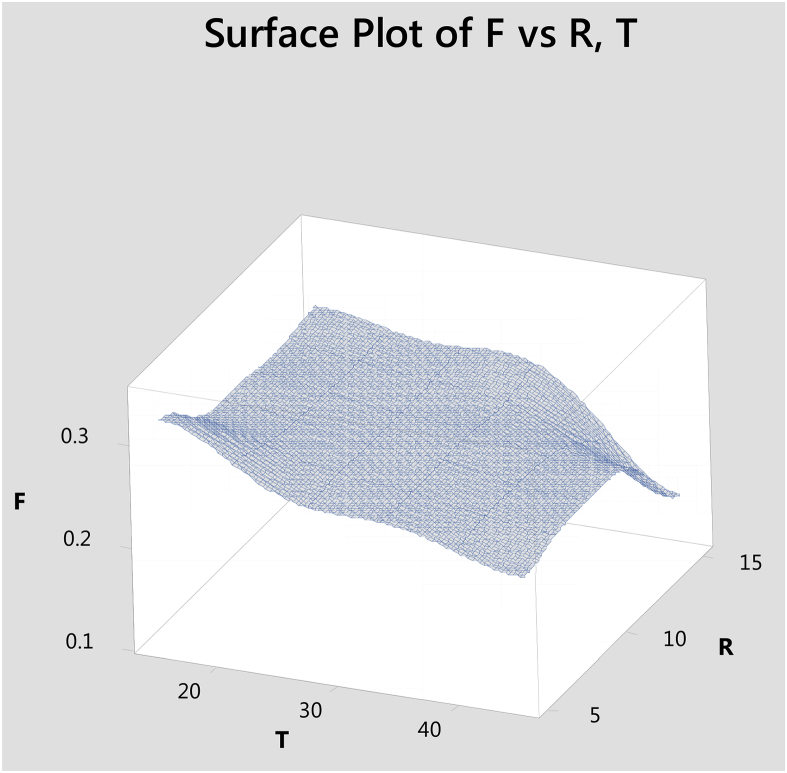
Surface plot to predict F with reference to T,R.

Surface plot to predict frictional values simultaneously with indication to the sliding time(T), and reinforcement(R) is given in Fig. 10. It shows with increasing the sliding time and reinforcement, the coefficient of friction decreases. Using "MINITAB 18″ software these plots are drawn.

**Fig. 11:**
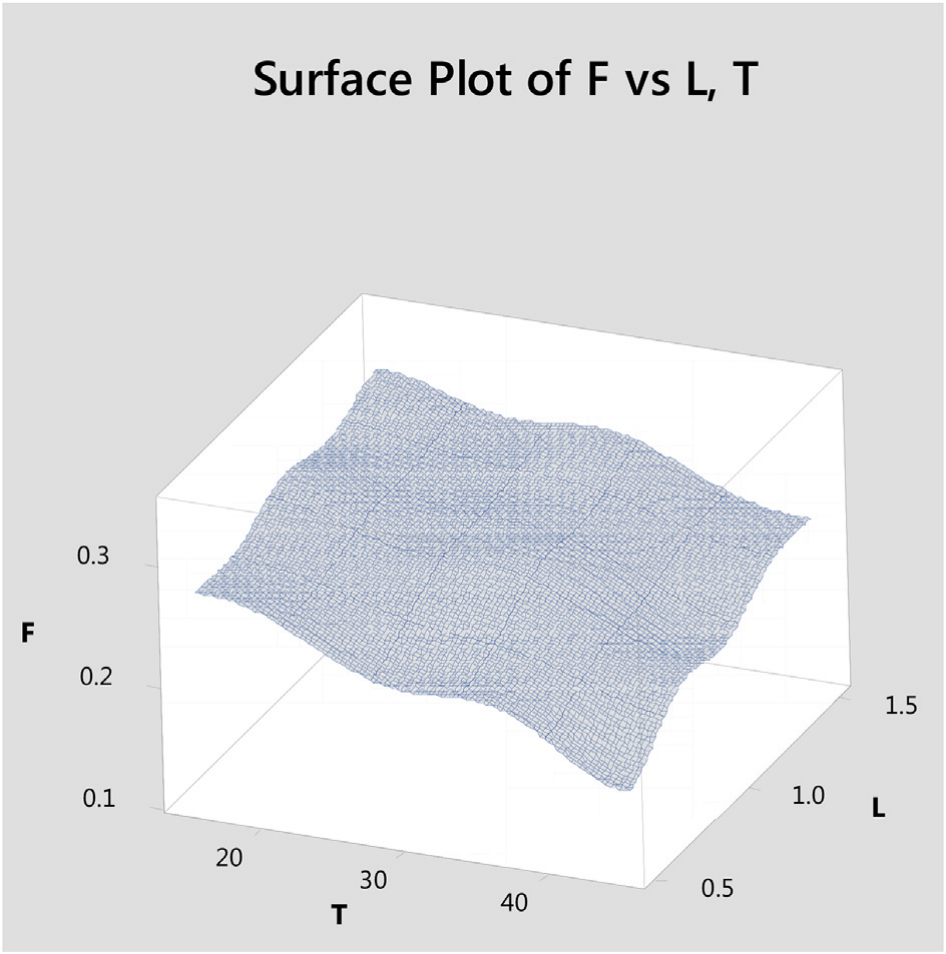
Surface plot to predict F with reference to T,L.

**Fig. 12:**
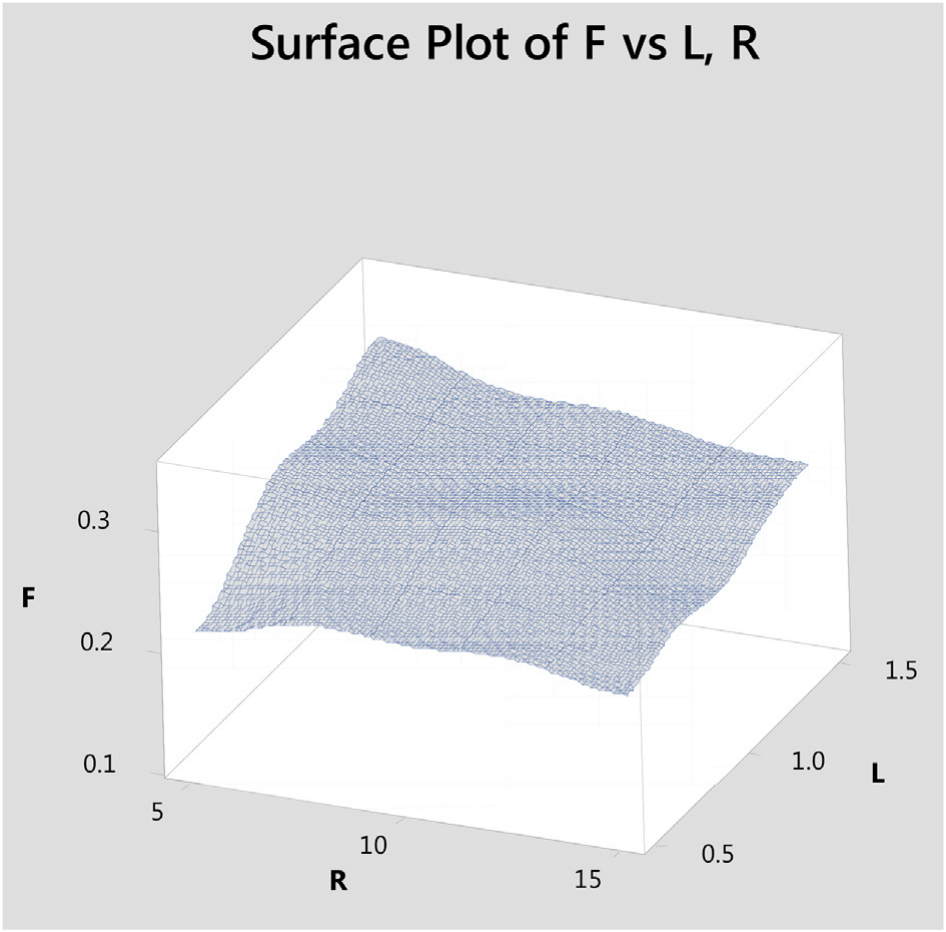
Surface plot to predict F with reference toR,L.

Surface plot to predict Frictional values simultaneously with reference to sliding time (T), Load (L) is given Fig. 11. Surface plot to predict Frictional values simultaneously with reference to reinforcement (R), Load (L) is given Fig. 12.

### 3.7 Examination of test specimens

**Fig. 13:**
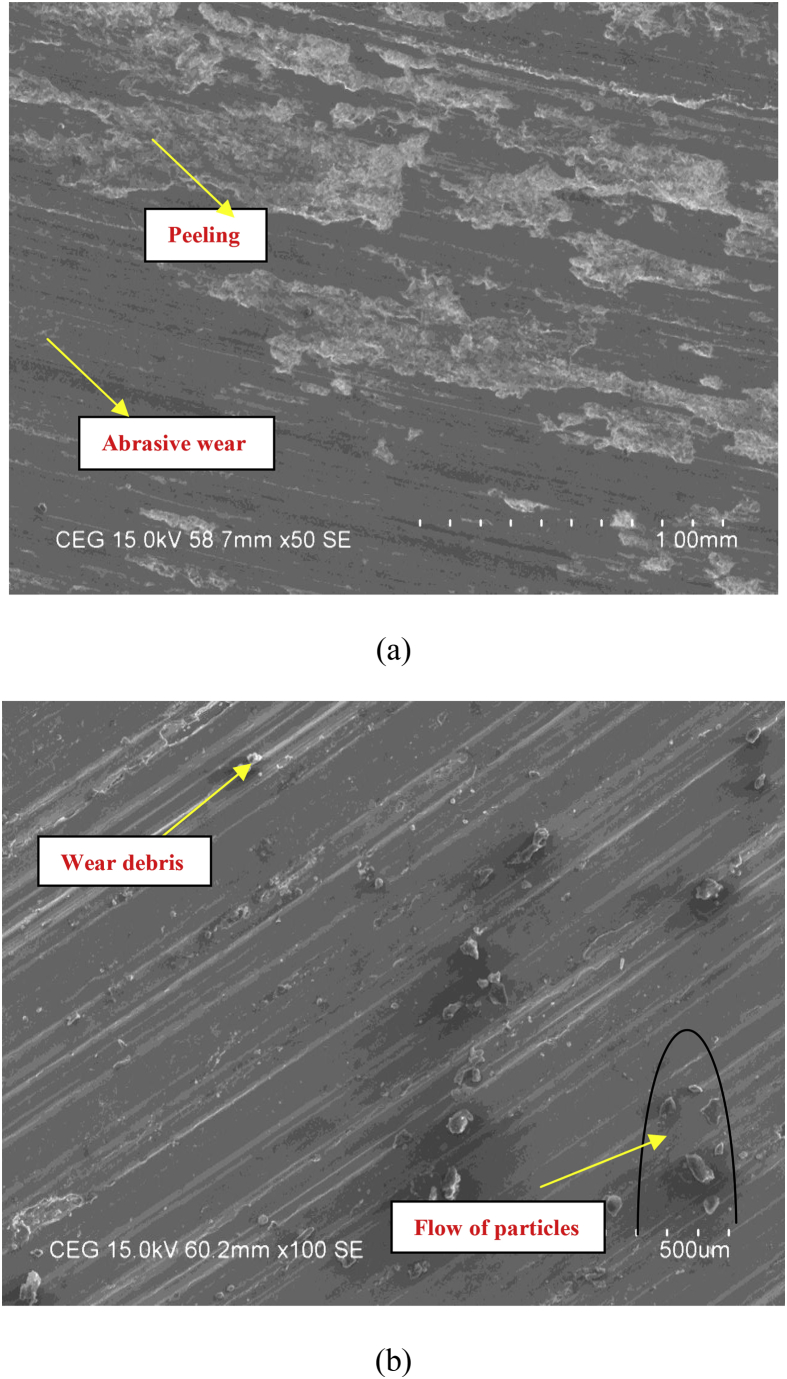
SEM image of worn surfaces at 1.5 kgf applied load and 2.7 km sliding. distance (a) alloy (b) composite.

The worn surfaces were examined and analyzed under SEM to comprehend the wear behavior after the wear test. The micrographs taken from SEM indicate that abrasive wear mechanism involved during wear test. Peeling is observed on wear surface indicate the involvement of abrasive wear mechanism with increase in loads as shown in Fig. 13 a. similar results were observed by Hassan, *et al.* [12] reported an increase in reinforcement content led to an increase in wear resistance and vice versa, indicating that the predicted model is accurate. The increase in wear resistance may be attributed to the strong interfacial bonding strength between the matrix and reinforcement resisting particle pull-out. The flow of particles on the wear surface is depicted in Fig. 13 b, with a little bit debris. The similar results were reported by several authors [8, 13, 14, 15, 16, 17, 18, 19, 20].

## 4 Conclusions

Hybrid aluminium metal matrix composites were tested for the dry sliding wear behavior by using pin on disc apparatus at room temperature, from the above analysis and results the following are the conclusions made.

1 The coefficient of friction of Graphite and granite dust reinforced 356 aluminium alloy hybrid composite decrease with increasing the reinforcement of particles and increasing the sliding time and increases with increase in sliding time.
2 The loading can be applied using force sensor by measuring the sliding and frictional force between the tribo-couple (composites pin and counter face steel disc).
3 The results of S/N ratio indicate that sliding time is a significant parameter on the coefficient of friction followed by load and Reinforcement.
4 From the ANOVA results, the sliding time (48.51%) is the most important contributing factor, followed by load (21.93%) and finally the percentage of the weight of Graphite and granite dust particles (7.01%) in coefficient of friction of studied hybrid composite.
5 Using Normal plot of the standardized effects it is represented the influence of parameters and its level of significance. 3D surface plots are drawn with the help of "MINITAB18″ software for simultaneously verifying the values of coefficient of friction with reference to any two sliding parameters.
6 SEM images of Worn out surfaces are verified and found various modes of Wear in terms of abrasive Mechanism, formation of oxide layer and its disintegration. With increase in loads and reinforcement contents grooves were found along with some abrasion layers but the severe delaminating effects were reduced.
7 The presence of particles flow on the wear surface and the traces of wear debris is identified in the SEM micrograph for composite.

## Declarations

### Author contribution statement

T.Satyanarayana: Conceived and designed the experiments; Performed the experiments; Contributed reagents, materials, analysis tools or data.

Putti Srinivasa Rao: Analyzed and interpreted the data; Contributed reagents, materials, analysis tools or data.

M. Gopi Krishna: Performed the experiments; Contributed reagents, materials, analysis tools or data; Wrote the paper.

### Funding statement

This research did not receive any specific grant from funding agencies in the public, commercial, or not-for-profit sectors.

### Competing interest statement

The authors declare no conflict of interest.

### Additional information

No additional information is available for this paper.
